# Bi-Anodal Transcranial Direct Current Stimulation Combined With Treadmill Walking Decreases Motor Cortical Activity in Young and Older Adults

**DOI:** 10.3389/fnagi.2021.739998

**Published:** 2021-12-03

**Authors:** Diego Orcioli-Silva, Aisha Islam, Mark R. Baker, Lilian Teresa Bucken Gobbi, Lynn Rochester, Annette Pantall

**Affiliations:** ^1^Institute of Biosciences, São Paulo State University (UNESP), Rio Claro, Brazil; ^2^Graduate Program in Movement Sciences, São Paulo State University (UNESP), Rio Claro, Brazil; ^3^Translational and Clinical Research Institute, Newcastle University, Newcastle upon Tyne, United Kingdom

**Keywords:** non-invasive brain stimulation, functional near-infrared spectroscopy, locomotion, cognition, ageing

## Abstract

**Background:** Walking in the “real world” involves motor and cognitive processes. In relation to this, declines in both motor function and cognition contribute to age-related gait dysfunction. Transcranial direct current stimulation (tDCS) and treadmill walking (STW) have potential to improve gait, particularly during dual-task walking (DTW); walking whilst performing a cognitive task. Our aims were to analyze effects of combined anodal tDCS + STW intervention on cortical activity and gait during DTW.

**Methods:** Twenty-three young adults (YA) and 21 older adults (OA) were randomly allocated to active or sham tDCS stimulation groups. Participants performed 5-min of mixed treadmill walking (alternating 30 s bouts of STW and DTW) before and after a 20-min intervention of active or sham tDCS + STW. Anodal electrodes were placed over the left prefrontal cortex (PFC) and the vertex (Cz) using 9 cm^2^ electrodes at 0.6 mA. Cortical activity of the PFC, primary motor cortex (M1), premotor cortex (PMC), and supplementary motor area (SMA) bilaterally were recorded using a functional near-infrared spectroscopy (fNIRS) system. Oxygenated hemoglobin (HbO_2_) levels were analyzed as indicators of cortical activity. An accelerometer measured gait parameters. We calculated the difference between DTW and STW for HbO_2_ and gait parameters. We applied linear mixed effects models which included age group (YA vs. OA), stimulation condition (sham vs. active), and time (pre- vs. post-intervention) as fixed effects. Treadmill belt speed was a covariate. Partial correlation tests were also performed.

**Results:** A main effect of age group was observed. OA displayed higher activity bilaterally in the PFC and M1, unilaterally in the right PMC and higher gait variability than YA. M1 activity decreased in both YA and OA following active tDCS + STW. There was no overall effect of tDCS + STW on PFC activity or gait parameters. However, negative correlations were observed between changes in left PFC and stride length variability following active tDCS + STW intervention.

**Conclusion:** Increased activity in multiple cortical areas during DTW in OA may act as a compensatory mechanism. Reduction in M1 activity following active tDCS + STW with no observed gait changes suggests improved neural efficiency.

## Introduction

Walking ability is a sensitive indicator of health status in older adults (OA) (Studenski et al., 2011; Morris et al., 2016). Gait dysfunction is common in OA which decreases independence, heightens falls risk (Lord et al., 1996; Hausdorff et al., 2001; Verghese et al., 2009), increases health care costs (Heinrich et al., 2009), and results in an overall decreased quality of life (Lin et al., 2015). Age-related gait changes linked with increased falls risk include reduced gait speed and step length, and increased gait variability in comparison to young adults (YA) (Hausdorff et al., 2001; Verghese et al., 2009; Sawa et al., 2014; Aboutorabi et al., 2016). These gait parameters have also been associated with deficits in cognitive parameters such as executive function and attention in OA and are a potential indicator of cognitive impairment (Yogev-Seligmann et al., 2008; Morris et al., 2016). The interplay between cognition and gait is frequently assessed in the laboratory with a dual-task walking (DTW) paradigm: walking whilst simultaneously conducting a cognitive task. DTW attempts to replicate features of “real world” walking when a person walks whilst performing additional tasks. Age-related gait impairments have been reported to be more pronounced during DTW, referred to as cognitive-motor interference [i.e., difference between DTW and single-task walking (STW)] (Al-Yahya et al., 2011; Plummer-D’Amato et al., 2012). These findings emphasize the need to investigate strategies for gait rehabilitation in OA during DTW.

Physical training may improve mobility, cognition and promote functional and structural brain adaptations (Steinberg et al., 2018; El-Sayes et al., 2019). Additional interventions, such as transcranial direct current stimulation (tDCS), may also enhance mobility and cognition (Fregni et al., 2005; Javadi and Walsh, 2012; Zhou et al., 2014; Hurley and Machado, 2018; Manor et al., 2018; Steinberg et al., 2018). tDCS is a low-cost method of non-invasive brain stimulation involving the application of low-amplitude currents over cortical regions of interest to modulate cortical excitability, but insufficient to generate action potentials (Nitsche and Paulus, 2000; Nitsche et al., 2008). Briefly, a direct current device delivers low current (0.5–2 mA) through anodal (positive) and cathodal (negative) electrodes placed at specific locations on the scalp (Nitsche et al., 2008; Brunoni et al., 2012; Hurley and Machado, 2018). Anodal tDCS results in depolarization and cathodal tDCS in hyperpolarization of resting membrane potential, leading to increased neuronal excitability or reduced neuronal excitability, respectively (Nitsche and Paulus, 2000; Nitsche et al., 2008; Hurley and Machado, 2018). Previous studies have shown that anodal tDCS causes enhancement of neural activity, which can result in improvement of motor control and cognitive function (Fregni et al., 2005; Nitsche et al., 2008; Brunoni et al., 2012; Hurley and Machado, 2018; Manor et al., 2018). Since physical training and anodal tDCS can each independently improve gait and cognitive performance, applying both simultaneously may enhance outcomes and prolong effects (Fregni et al., 2005; Steinberg et al., 2018). Studies have demonstrated that acute physical training combined with anodal tDCS beneficially modifies gait parameters and cognition (Kaski et al., 2014b,a; Park et al., 2015; Manenti et al., 2016; Ishikuro et al., 2018). However, recent reviews of the literature (de Paz et al., 2019; Beretta et al., 2020) on the efficacy of combining physical training with anodal tDCS on gait were inconclusive. For example, a total of seven studies (Costa-Ribeiro et al., 2016, 2017; Kumru et al., 2016; Manenti et al., 2016; Fernández-Lago et al., 2017; Seo et al., 2017; Yotnuengnit et al., 2018) did not observe an improvement in walking performance after physical training combined with tDCS in patients with neurological disorders. The interpretation of such studies is limited by the fact that most previous studies applied tDCS over a single cortical area, typically either over the primary motor cortex (M1) or prefrontal cortex (PFC) (de Paz et al., 2019; Beretta et al., 2020). The efficacy of tDCS combined with physical training in gait rehabilitation therefore remains uncertain.

M1 and PFC play an important and specific role during walking. M1 is involved in the execution of movements (control of lower limb and trunk muscles) related to walking (Petersen et al., 2001, 2012), whilst the PFC has a modulatory function in the allocation of attention during gait (Koenraadt et al., 2014). In addition, M1 is the main contributor to the direct locomotor pathway, which is activated in the absence of pathologies or challenging situations (la Fougère et al., 2010; Herold et al., 2017). PFC is involved in the indirect locomotor pathway, which contributes more to gait control when the direct locomotor pathway is impaired, even during single-task walking (STW) (la Fougère et al., 2010; Herold et al., 2017). Studies assessing cortical activity using functional near-infrared spectroscopy (fNIRS) during different walking tasks provide evidence for the different roles of M1 and PFC. Previous studies have demonstrated that OA have higher prefrontal cortex (PFC) activity during STW compared to YA (Herold et al., 2017; Vitorio et al., 2017; Stuart et al., 2018; Pelicioni et al., 2019; Nóbrega-Sousa et al., 2020), which is increased during DTW (Herold et al., 2017; Vitorio et al., 2017; Stuart et al., 2018; Pelicioni et al., 2019; Nóbrega-Sousa et al., 2020). The increased PFC activity is theorized to be a cognitive compensation for age-related deficits (Cabeza et al., 2002; Bierre et al., 2017; Machado, 2021), recruiting additional cognitive resources, such as increased attention, during walking (Cabeza et al., 2002). Previous studies have indicated that increased M1 activity improves gait parameters (Koganemaru et al., 2018) and increased PFC activity improves cognitive function (Yanagisawa et al., 2010; Byun et al., 2014; Ji et al., 2019). Thus, M1 stimulation may facilitate the movement execution and PFC stimulation may promote greater cognitive resources for the task, which suggests that stimulation of both cortical areas may improve DTW performance.

In this study, we aimed to analyze the effect of a combined anodal tDCS (M1 and PFC stimulation) and treadmill walking intervention (tDCS + STW) on cortical activity (as measured by fNIRS) and gait parameters during DTW in YA and OA. As anodal tDCS is considered to increase excitability and facilitate the functional activation of M1 and PFC (Nitsche and Paulus, 2000), we hypothesized that activity in these areas would increase in both age groups during DTW following the anodal tDCS + STW intervention, but no such increases would occur in the control groups (following sham tDCS + STW). We also expected treadmill gait parameters during DTW in both OA and YA to improve (e.g., reduced gait variability) following the active tDCS + STW intervention but not in the control groups (following sham tDCS + STW), with greater benefits in OA, due to this group having greater gait impairments (Hausdorff et al., 2008; Yogev-Seligmann et al., 2008; Beurskens and Bock, 2012).

## Materials and Methods

This study used a double-blinded, randomized, and sham-controlled design. Ethical approval was granted by Newcastle University (ref. 6770/2018). We performed a power analysis, using data from a previous study investigating age-related differences in cortical activity, to determine the sample size of 7 necessary to detect a difference in HbO_2_ of 12% with a standard deviation of 7% and power of 0.8 (Vitorio et al., 2018). An increase of over 38% in HbO_2_ levels in the PFC has previously been reported following the application of single anodal tDCS to the PFC (da Conceição et al., 2021). A minimum sample size of 7 per group is therefore sufficient to detect anticipated changes in HbO_2_ following administration of tDCS.

**FIGURE 1:**
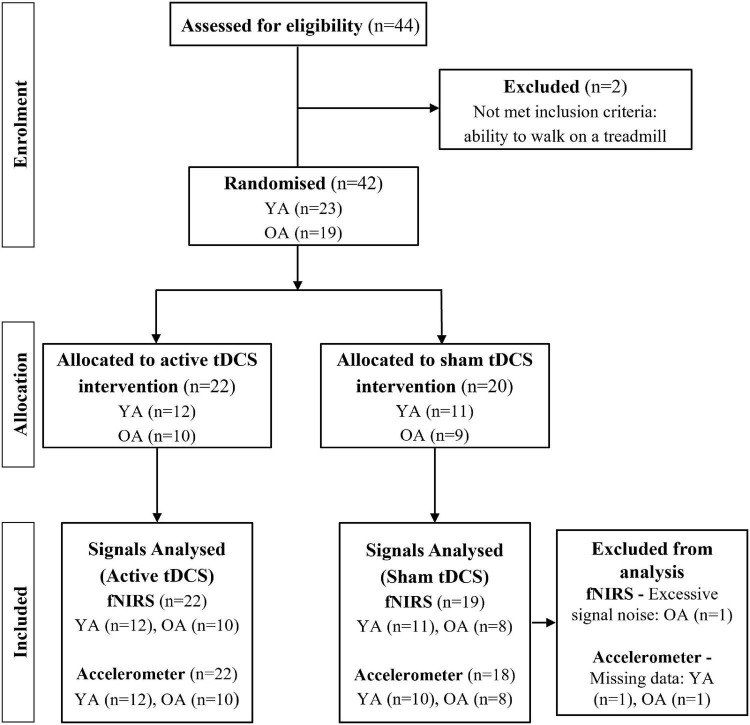
Participant recruitment flowchart. YA: young adults; OA: older adults; tDCS: transcranial direct current stimulation; fNIRS: functional near-infrared spectroscopy.

Forty-four participants were recruited and assigned into two groups: healthy young adults (YA; *n* = 23) and healthy older adults (OA; *n* = 21). Prior to the experiment, OA and YA were randomly allocated to active tDCS intervention (active-OA and active-YA) or sham tDCS intervention (sham-OA and sham-YA) (Figure 1). The inclusion criteria were YA aged between 18–40 years and OA aged ≥60 years, able to walk unaided for 5-min and good English language comprehension. Exclusion criteria included cognitive impairment [Montreal Cognitive Assessment (MoCA) score ≤ 21], psychiatric co-morbidities, history of drug or alcohol abuse, chronic musculoskeletal, cardiovascular or respiratory disease affecting gait, implanted metal objects, and a history of seizures or any contraindication to tDCS. This study was conducted according to the declaration of Helsinki and all participants signed an informed consent form prior to testing.

### Study Design

Demographic characteristics and cognitive status were obtained for all participants at the beginning of the experiment. The MoCA was used to determine global cognitive function (Nasreddine et al., 2005). Fear of falling was assessed with the Falls Efficacy Scale - International (FES-I) score (Yardley et al., 2005). Participants also reported how many hours a week they exercised.

Participants performed two bouts of 5-min mixed treadmill walking before and after 20-min of tDCS + STW at self-selected speeds. We used the block design, adhering to previous recommendations for fNIRS studies (Herold et al., 2017; Vitorio et al., 2017). The 5-min mixed treadmill walking consisted of 10 trials of alternating 30 s STW and 30 s DTW bouts. The self-selected treadmill speed was maintained during the entire experiment and was determined by increasing belt speed until it was faster than the participants’ preferred speed, then reducing belt speed until preferred speed was achieved (Vitorio et al., 2018). This was conducted whilst participants were blinded to their walking speed. DTW consisted of a digit vigilance task, which required participants to walk while listening to random numbers (from 1 to 9) played over a loudspeaker for 30 s. The intervals between numbers were randomized to prevent gait synchronization. Following cessation of the numbers, participants stated how many odd or even numbers they had heard. Speech was minimized to prevent motion artifact contaminating the fNIRS signals. Immediately before walking commenced, participants were given the class of numbers (odd or even) they were required to count. The performance in the cognitive digit vigilance task was quantified by the absolute error (difference between the correct answer and the response given by the participant) and expressed in percentage (0% indicates that there is no error).

### Transcranial Direct Current Stimulation and Treadmill Walking Intervention

**FIGURE 2:**
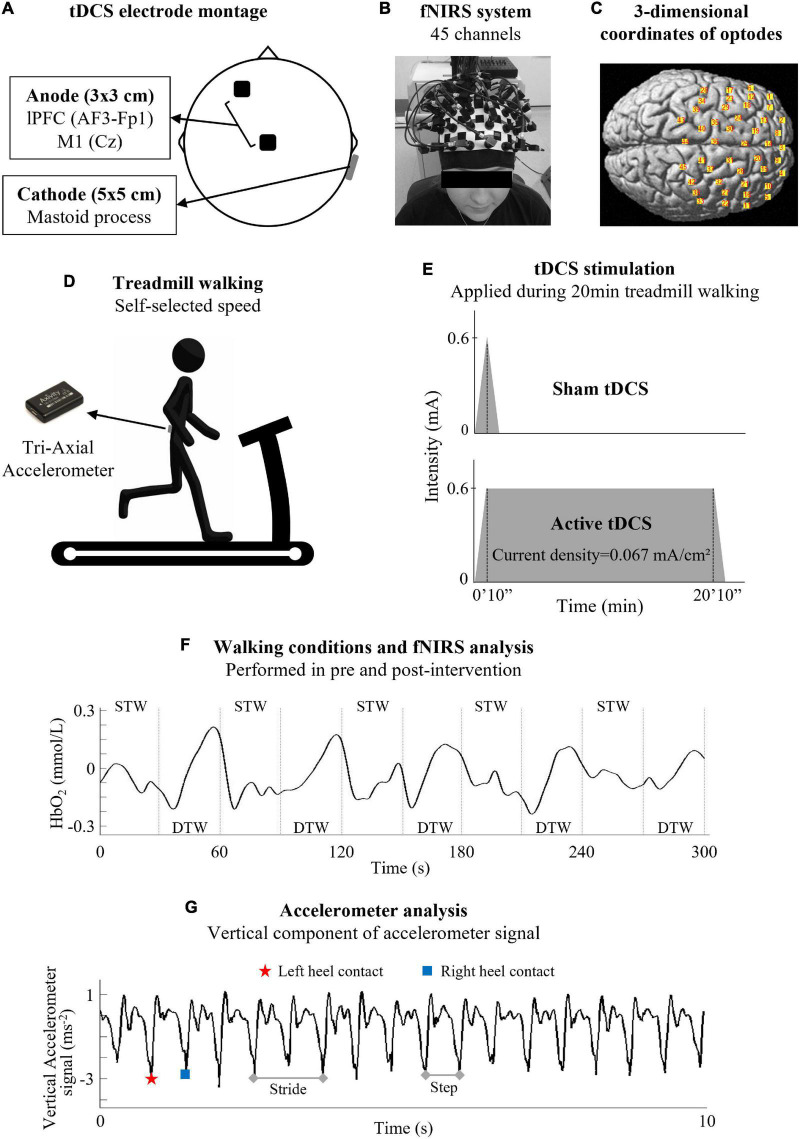
Experimental setup. **(A)** Transcranial direct current stimulation (tDCS) was positioned over the left prefrontal cortex (lPFC) and Cz (i.e., the vertex, which overlies the primary motor cortex - M1), following the 10/20 EEG system. After tDCS positioning, **(B)** a headcap with fNIRS optodes was positioned on the participants’ head. **(C)** Spatial registration of the 45-channels was calculated using a digitizer (FASTRAK) to confirm the optode position. **(D)** A tri-axial accelerometer was positioned over the 5th lumbar vertebra. Then, participants performed two bouts of 5-min mixed treadmill walking before and after 20-min treadmill walking combined with tDCS protocol. **(E)** Participants were randomly allocated to active tDCS intervention and received a 0.6 mA stimulation for 20-min, or sham tDCS intervention, and received a 0.6 mA stimulation for only 10 s. **(F)** The 5-min mixed treadmill walking consisted of 10 trials of alternating 30 s for both single task walking (STW) and dual task walking (DTW) bouts. HbO_2_ concentration from the STW was subtracted from the DTW to evaluate the relative change in HbO_2_ concentration (Δ_DTW–STW_). **(G)** Gait cycles were calculated using the accelerometer and the cognitive-motor interference was also calculated (difference between DTW and STW).

The experimental setup is summarized in Figure 2. Participants performed a total of 20-min of single-task treadmill walking at self-selected speed combined with anodal tDCS. Only the experimenter that applied the tDCS was aware of the intervention allocation of the individual (active or sham) to ensure both the participant and other experimenters were blinded. The active group received anodal tDCS over Cz (i.e., the vertex, which overlies M1) and the left PFC, between AF3 to Fp1 (9 cm anterior and 3 cm lateral to Cz), on the 10/20 EEG system, using a 3 × 3 cm^2^ electrode. The cathode (5 × 5 cm^2^) was positioned over the right mastoid, contralateral to the left PFC (Figure 2A). We selected the left PFC because tDCS applied to this area acutely was observed to improve both cognitive (Andrews et al., 2011; Chrysikou et al., 2013; Vanderhasselt et al., 2013) and motor functions (Wrightson et al., 2015; Manor et al., 2018; Schneider et al., 2021). tDCS was applied using a battery-driven constant current stimulator (HDCStim, Newronika, Italy) with conductive paste to affix the electrodes to the scalp. tDCS was delivered at 0.6 mA for 20-min with a ramp-up of 10 s. We chose 0.6 mA because we used small tDCS electrodes (area 9 cm^2^). Thus, we decreased the intensity of the current to ensure that the current density (current strength divided by electrode size) was maintained at 0.067 mA/cm^2^, within the recommended safety limits (0.029–0.08 mA/cm^2^) (Nitsche et al., 2008). In the sham stimulation, the tDCS montage was the same, but the current ramped down 10 s after the beginning of stimulation. This procedure provided a similar sensation of active stimulation but did not induce neurophysiological changes (Nitsche et al., 2008). At the end of the experiment, the participants completed an adverse events questionnaire to monitor differences in the perception of the stimulation experienced during active and sham tDCS (Brunoni et al., 2011). The rating of perceived exertion scale (Borg scale) was applied at the beginning, middle and end of the intervention period (0, 10, and 20 min, respectively) to measure the participant’s effort and exertion.

### Functional Near-Infrared Spectroscopy Recordings and Processing

After tDCS positioning, a headcap with fNIRS optodes was positioned on the participants’ head. Both the fNIRS system and tDCS electrodes remained in place during the entire experimental protocol. We did not remove the fNIRS system during the intervention to ensure the consistency in the brain regions sampled pre- and post-intervention. Changes in oxygenated (HbO_2_) and deoxygenated hemoglobin (HHb) were recorded with a sampling frequency of 22.2 Hz using a tethered fNIRS optical imaging system (LABNIRS; Shimadzu, Kyoto, Japan), with continuous wave laser diodes with wavelengths of 780, 805, 830 nm. The optical density of the raw signal was converted into HbO_2_ and HHb using a modified Beer-Lambert Law. A 45-channel arrangement with 24 fiber optic optodes, consisting of 12 transmitters and 12 detectors, covered both hemispheres of the frontal lobe (Figures 2B–C). Emitter-detector distance was 30 mm. Participants wore a custom-made whole-head optode holder marked according to the international 10–20 EEG System (Figure 2B). A digitizer (FASTRAK, Polhemus, VT, United States) was used to register 3-dimensional coordinates of optodes and stimulation sites relative to landmarks (nasion, Cz, left and right pre-auricular points). The spatial registration was calculated using the free software package NIRS-SPM (Ye et al., 2009), which allows registration of fNIRS channel data onto the Montreal Neurological Institute standard space (Tsuzuki and Dan, 2014; Figure 2C). The brain regions of interest (ROI) measured included PFC (Brodmann areas 8, 9, 10, 45, and 46), PMC (Brodmann area 6, lateral), SMA (Brodmann area 6, medial), and M1 (Brodmann area 4) (Vitorio et al., 2018).

Processing of fNIRS followed previous recommendations (Vitorio et al., 2017). We selected the HbO_2_ concentration as it is the most sensitive indicator of walking-related changes in cortical activity (Suzuki et al., 2004; Harada et al., 2009). The fNIRS data were pre-processed using NIRS-SPM open source toolbox for MATLAB (Ye et al., 2009). A low-pass filter (cut-off 0.14 Hz) based on a canonical hemodynamic response function was used to reduce the high-frequency noise (Friston et al., 2000). A wavelet-minimum description length detrending algorithm was applied to decompose NIRS measurements into global trends, hemodynamic signals, and uncorrelated noise components as distinct scales (Jang et al., 2009). Pre-processed data were exported to MATLAB (MATLAB and Statistics Toolbox Release 2015a, The MathWorks, Inc., Natick, MA, United States), in which further data processing was performed using customized scripts. Firstly, HbO_2_ concentration signals were averaged per ROI (right and left PFC, PMC, SMA, and M1) and normalized by dividing them by corresponding signal amplitude (from minimum to maximum) value during the mixed treadmill walking (Koenraadt et al., 2014; Vitorio et al., 2018; Orcioli-Silva et al., 2020, 2021). Then, data were divided into two phases (Figure 2F): (i) a period running from 5 to 25 s of STW and (ii) a period running from 5 to 25 s of DTW. The initial 5 s and final 5 s of the tasks were removed due to the hemodynamic response phase lag (Vitorio et al., 2018). Subsequently, the normalized HbO_2_ concentration was averaged (in time) over the STW (20 s) and DTW periods (20 s) for each ROI and each trial. Normalized HbO_2_ concentration from STW was subtracted from the DTW to evaluate the relative change in HbO_2_ concentration (Δ_DTW–STW_) (Maidan et al., 2016; Mirelman et al., 2017; Vitorio et al., 2018; Nóbrega-Sousa et al., 2020; Orcioli-Silva et al., 2020, 2021). The fNIRS outcome measure, ΔHbO_2_, therefore represents the change in cortical activity during DTW compared to STW.

### Gait Parameters Recordings and Processing

A tri-axial accelerometer (Axivity Ltd., Newcastle upon Tyne, United Kingdom), sampling at 100 Hz, positioned over the 5th lumbar vertebra, recorded trunk acceleration during the 5-min mixed treadmill walking before and after the intervention (Figure 2D). Gait parameters were extracted from the accelerometry data using previously validated algorithms (Del Din et al., 2016). Briefly, acceleration data were transformed to a horizontal-vertical coordinate system (Moe-Nilssen, 1998) and filtered with a fourth-order Butterworth filter (20 Hz) (Zijlstra and Hof, 2003; McCamley et al., 2012). Initial and final contact events within the gait cycle were estimated with a continuous wavelet transform (CWT) of the vertical acceleration which was first integrated and then differentiated using a Gaussian CWT. The initial and final contact events were detected as the local minima and maxima of the CWT, respectively (Del Din et al., 2016; Figure 2G). Both right and left heel strike were identified. Initial contact and final contact detection times were used to estimate the step, stance time (Del Din et al., 2016). Step/stride length was determined from the initial contact events through application of the inverted pendulum model described by Zijlstra and Hof (2003). We chose gait parameters that have been previously related to falls, such as the stance time ratio (Verghese et al., 2009), cadence (Lord et al., 1996), stride time variability (Hausdorff et al., 2001), and stride length variability (Verghese et al., 2009). We calculated the gait variability using the standard deviation from all steps (Del Din et al., 2016). Stance time ratio, also referred to as duty factor, is the ratio between the foot contact time and the stride time (Voigt et al., 2019). This parameter has important links to motor control system dynamics as well as to muscle metabolic energy expenditure (Beck et al., 2020). Gait speed was a covariate because we used a fixed treadmill speed for each individual. The difference between DTW and STW (Δ_DTW–STW_) for these selected gait parameters, which represents the cognitive-motor interference, was also calculated (Al-Yahya et al., 2011).

### Statistical Analysis

Statistical analysis was performed using SPSS (v22, IBM, Armonk, NY, United States) for Windows. The level of significance was set at *p* ≤ 0.05. Characterization data were analyzed using two-way ANOVAs with age group (YA and OA) and stimulation condition (active vs. sham tDCS) as independent variables. Chi-square test was applied to compare difference in sex between age groups or stimulation condition. The Borg scale was analyzed using linear mixed effects models with age group, stimulation condition, intervention duration (0, 10, and 20 min), and interactions as fixed effects. Differences in DTW related changes in gait, HbO_2_ per ROI, and cognitive task were analyzed using linear mixed effects models. Fixed effects included were age group, stimulation condition, and time (pre- vs. post-intervention) with treadmill speed as a covariate. *Post hoc* tests with Bonferroni adjustment were used to localize the differences in significant main effects or interactions. Partial correlation tests were calculated separately for active and sham groups to explore the associations between gait parameters and cortical activity in response to intervention (Δ_POST–PRE_), while controlling for treadmill velocity and age. The partial eta-squared (η^2^_p_: 0.01 = small, 0.06 = moderate, 0.14 = large) and Cohen’s *d* (*d*: 0.2 = small, 0.5 = moderate, 0.8 = large) statistic provided estimates of the effect sizes.

## Results

The characteristics of the participants are summarized in Table 1. The two-way ANOVA revealed a main effect of age group for body mass [*F*_(1,38)_ = 27.37, *p* = 0.045, η^2^_p_ = 0.113], preferred treadmill speed [*F*_(1,38)_ = 27.37, *p* < 0.001, η^2^_p_ = 0.419] and the Adverse Effects of tDCS Questionnaire [*F*_(1,38)_ = 16.05, *p* < 0.001, η^2^_p_ = 0.297]. OA had higher body mass compared to YA (77.0 ± 4.4 kg; 64.3 ± 4.2 kg); OA walked at a slower treadmill speed than YA (2.71 ± 0.17 ms^–2^; 3.87 ± 0.15 ms^–2^), OA showed lower scores (fewer adverse effects) on the Adverse Effects of tDCS Questionnaire than YA (10.58 ± 0.55; 13.56 ± 0.50). There was no significant effect of stimulation condition.

**TABLE 1:** Participant characteristics (Mean ± SD).

	Older adults		Young adults	
	Active (*n* = 10)	Sham (*n* = 9)	Active (*n* = 12)	Sham (*n* = 11)
Age (years)*	66.0 ± 6.3	69.9 ± 4.8	19.3 ± 1.1	20.9 ± 4.2
Male/Female	5/5	2/7	1/11	2/9
Height (cm)	171.6 ± 10.7	167.7 ± 11.3	167.6 ± 7.7	174.4 ± 9.7
Body mass (kg)*	71.0 ± 9.4	73.6 ± 8.3	62.8 ± 10.0	65.89 ± 11.9
Education (years)	16.9 ± 2.9	15.7 ± 4.0	15.2 ± 1.0	16.3 ± 2.4
MoCA (0–30)	28.2 ± 1.1	28.3 ± 1.6	28.4 ± 2.1	28.8 ± 1.4
FES-I (16–64)	17.6 ± 1.2	18.1 ± 0.9	18.5 ± 2.1	18.6 ± 3.0
Exercise (hours/week)	8.2 ± 5.6	11.2 ± 5.9	7.3 ± 4.0	6.3 ± 6.5
Treadmill Speed (Km/h)*	2.9 ± 0.8	2.5 ± 1.0	4.0 ± 0.4	3.7 ± 0.7
AE Questionnaire (10–40)*	10.5 ± 0.7	10.7 ± 1.3	13.4 ± 1.7	13.7 ± 4.3

MoCA, Montreal Cognitive Assessment; FES-I, Falls Efficacy Scale International; AE Questionnaire, Adverse Events Questionnaire. *: Significant effect of Age Group p < 0.05.

**FIGURE 3:**
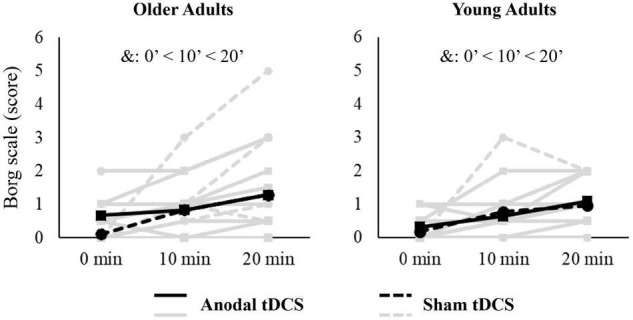
Individuals (light gray lines) and means data (black lines) of perceived exertion scale (Borg) applied during 20-min treadmill walking combined with tDCS. &: indicates a main effect of intervention duration.

The linear mixed effects models showed a main effect of intervention duration (0, 10, and 20 min) for the Borg scale [*F*_(2,108)_ = 10.398, *p* < 0.001, η^2^_p_ = 0.161] (Figure 3). The perceived exertion increased throughout the tDCS + STW intervention with the Borg scale score being higher in the 10th min compared to 0 min (*p* = 0.015, *d* = 0.728) and in the 20th min compared to 0 min (*p* < 0.001, *d* = 1.007) and 10th min (*p* = 0.039, *d* = 0.409).

Linear mixed effects models did not show main effects of age group, stimulation condition, and time, in addition to interaction effects for the performance (% of error) in the cognitive digit vigilance task during DTW (Table 2).

**TABLE 2:** Means and standard deviation of dual task interference on gait parameters (Δ_DTW–STW_) and cognitive task error for pre- and post-intervention.

	Older adults				Young adults			
Variables	Active tDCS (*n* = 10)		Sham tDCS (*n* = 8)		Active tDCS (*n* = 12)		Sham tDCS (*n* = 11)	
	Pre	Post	Pre	Post	Pre	Post	Pre	Post
Cadence (step/min)	−0.08 ± 3.24	1.64 ± 1.68	−0.30 ± 3.50	0.92 ± 1.16	−0.01 ± 2.42	0.13 ± 0.85	0.22 ± 0.88	0.64 ± 1.00
Stance time ratio^a^	−0.002 ± 0.004	0.004 ± 0.009	−0.006 ± 0.012	0.000 ± 0.007	−0.001 ± 0.007	−0.002 ± 0.003	0.001 ± 0.003	0.000 ± 0.002
Stride time variability (s)	0.024 ± 0.034	−0.010 ± 0.020	0.050 ± 0.048	0.010 ± 0.040	−0.005 ± 0.064	−0.003 ± 0.004	0.001 ± 0.007	0.001 ± 0.018
Stride length variability (s)	−0.011 ± 0.012	0.005 ± 0.021	0.004 ± 0.021	0.007 ± 0.018	−0.023 ± 0.025	−0.013 ± 0.019	−0.012 ± 0.014	−0.012 ± 0.016
Cognitive task errors (%)^b^	3.53 ± 4.07	3.98 ± 5.78	4.32 ± 5.10	3.68 ± 7.35	4.15 ± 4.69	1.05 ± 1.60	4.58 ± 7.25	2.93 ± 5.51

*^a^Ratio between the foot contact time and the stride time. ^b^Digit vigilance task.*

Data from some participants were excluded from analysis because of excessive fNIRS noise across all channels (one from sham-OA group), or because of problems with the accelerometer recordings (one from sham-OA group and one from sham-YA group). Hence, fNIRS analysis was based on *n* = 10 for the active-OA, *n* = 8 for the sham-OA, *n* = 12 for the active-YA, and *n* = 11 for the sham-YA. The accelerometer analysis was based on *n* = 10 for the active-OA, *n* = 8 for the sham-OA, *n* = 12 for the active-YA, and *n* = 10 for the sham-YA.

### Effect of Transcranial Direct Current Stimulation Combined With Treadmill Walking on ΔHbO_2_ Levels

**FIGURE 4:**
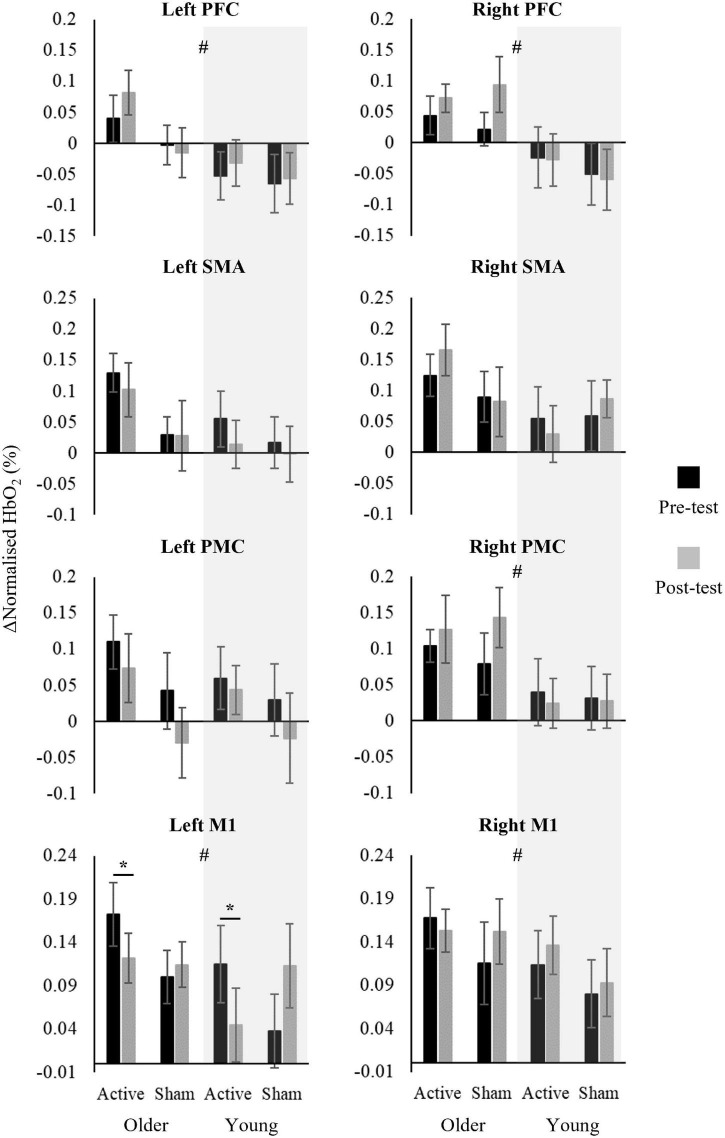
Means and standard errors of change in oxygenated hemoglobin (ΔHbO_2_ = DTW periods minus STW periods) in pre- and post-intervention walking test of active-Older adults (*n* = 10), sham-Older adults (*n* = 8), active-Young adults (*n* = 12) and sham-Young adults (*n* = 11). # indicates significant main effect of age group and * indicates significant interaction between stimulation condition and time.

The linear mixed effects models showed a main effect of age group, with OA presenting higher ΔHbO_2_ in the left PFC [*F*_(1,74)_ = 5.348, *p* = 0.024, η^2^_p_ = 0.067], right PFC [*F*_(1,74)_ = 11.859, *p* = 0.001, η^2^_p_ = 0.138], right PMC [*F*_(1,74)_ = 6.601, *p* = 0.012, η^2^_p_ = 0.082], left M1 [*F*_(1,74)_ = 4.579, *p* = 0.036, η^2^_p_ = 0.058], and right M1 [*F*_(1,74)_ = 4.084, *p* = 0.047, η^2^_p_ = 0.052] compared to YA (Figure 4). In addition, an interaction effect between stimulation condition and time was found for ΔHbO_2_ in the left M1 [*F*_(1,74)_ = 4.795, *p* = 0.032, η^2^_p_ = 0.061] (Figure 4). *Post hoc* test showed that both OA and YA receiving active tDCS decreased left M1 ΔHbO_2_ after the tDCS + STW intervention compared to pre-intervention (*p* = 0.040, *d* = 0.615). No other main effects of age group, stimulation condition or time, or interaction effects, were found.

### Effect of Age, Time, and Intervention on Dual-Task-Related Gait Changes

Gait parameters are presented in Table 2 and the summary of effects are presented in Table 3. A main effect of age group was observed for Δstride time variability [*F*_(1,72)_ = 6.011, *p* = 0.017, η^2^_p_ = 0.077] and Δstride length variability [*F*_(1,72)_ = 14.572, *p* < 0.001, η^2^_p_ = 0.168], with OAs presenting higher Δ values than YAs. There was a main effect of time for Δstride time variability [*F*_(1,72)_ = 4.985, *p* = 0.029, η^2^_p_ = 0.065], showing that the difference in stride time variability between DTW and STW (delta value) decreased in participants post-intervention compared to pre-intervention. An interaction between age group and time was found for Δstance time ratio [*F*_(1,72)_ = 4.798, *p* = 0.032, η^2^_p_ = 0.062] and Δstride time variability [*F*_(1,72)_ = 5.928, *p* = 0.017, η^2^_p_ = 0.076]. *Post hoc* tests showed higher Δstride time variability in pre-intervention for OA compared to YA (*p* = 0.001, *d* = 0.855). In addition, OA increased Δstance time ratio (*p* = 0.013, *d* = 0.645) and decreased Δstride time variability (*p* = 0.002, *d* = 1.001) in post-intervention compared to pre-intervention, while no change was observed for YA. No other main effects of age, stimulation condition or time, or interaction effects, were found.

**TABLE 3:** Summary of main effect and interactions in 3-way linear mixed models analyses of gait parameters.

Gait parameters	Main effect			Interactions	
	Age group^c^	Intervention group^d^	Time^e^	Age × Time	Intervention × Time
Cadence	ns	ns	ns	ns	ns
Stance time ratio^a^	ns	ns	ns	OA: Pre < Post	ns
Stride time variability	ns	ns	Pre > Post	Pre: OA > YA	ns
				OA: Pre > Post	
Stride length variability	ns	ns	ns	ns	ns
Cognitive task errors^b^	ns	ns	ns	ns	ns

*^a^Ratio between the foot contact time and the stride time. ^b^Digit vigilance task. ^c^Older adults (OA) vs. young adults (YA). ^d^Active tDCS + STW vs. Sham tDCS + STW. ^e^Pre- vs. post-intervention. ns: not significant.*

### Association Between Change in Cortical Activity and Gait Parameters in Response to Intervention (Δ_POST–PRE_)

**FIGURE 5:**
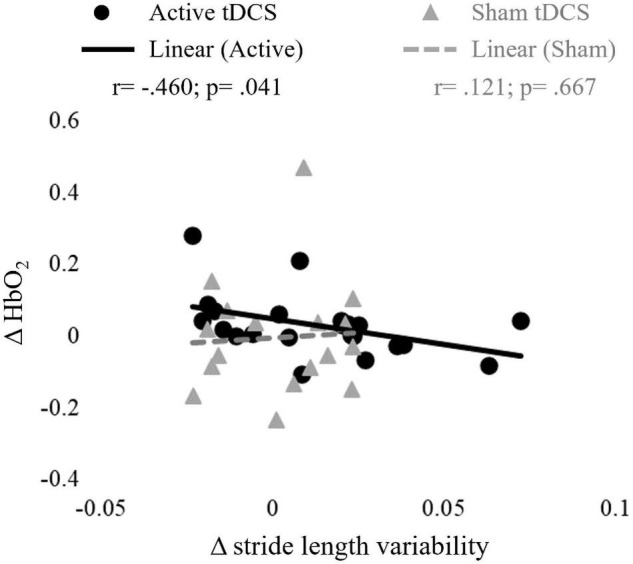
Correlations between changes in oxygenated hemoglobin (ΔHbO_2_) in the left prefrontal cortex (PFC) activity and stride length variability in responses to active and sham tDCS stimulation (Δ_POST–PRE_).

A negative correlation was observed between ΔHbO_2_ in the left PFC and Δstride length variability for active groups (Figure 5). There were no other significant associations between ΔHbO_2_ and changes in gait parameters that occurred in any of the groups.

## Discussion

In this study we investigated the effects of combined anodal tDCS applied over M1 and PFC, and treadmill walking on cortical activity and gait parameters in YA and OA. Contrary to our hypothesis, we found that active anodal tDCS + STW decreased M1 activity in both YA and OA and did not modify gait parameters. A negative correlation was observed between changes in PFC activity and stride length variability.

A novelty of this study was to apply tDCS over two brain areas, M1 and PFC as previous studies have applied the stimulation to these areas separately. These regions were selected as DTW involves both motor and higher executive function control (Petersen et al., 2001, 2012; Koenraadt et al., 2014). Our main findings showed that M1 ΔHbO_2_ decreased following the active tDCS + STW intervention with no change in PFC. Previous studies have indicated that anodal tDCS increases excitability in the target area, due to postulated increased action potential firing rates, prolonged changes in membrane potential and decreased inhibitory interneural activity (Nitsche and Paulus, 2000; Nitsche et al., 2008; Murray et al., 2015; Hurley and Machado, 2018). Therefore, we expected to observe an increase in PFC and M1 activity. A possible explanation, according to the neural efficiency hypothesis, is that anodal tDCS may have improved the efficiency of M1 activity (Zarahn et al., 2007). Increased cortical activity has been considered a compensatory strategy for maintaining motor performance (Herold et al., 2017; Stuart et al., 2018). However, reduced cortical activity without changing motor performance demonstrates an improvement in neural efficiency, that is, individuals with higher neural ability display a lower energy consumption of the brain (Zarahn et al., 2007). Indeed, both YA and OA did not change the locomotor pattern post-intervention. Taken together, our findings showed that anodal tDCS over M1 contributes to improving neural efficiency to control walking when performing a cognitive task simultaneously.

A further possible explanation for decreased M1 activity after active tDCS + STW intervention is homeostatic metaplasticity. Bienenstock et al. (1982) developed a mathematical model, the Bienenstock–Cooper–Munro (BCM) theory, to describe modulation of synaptic excitability based on homeostatic metaplasticity of synapses. Homeostatic metaplasticity is a mechanism that maintains neuronal excitability within a physiological dynamic range (Murakami et al., 2012). This theory postulates that plasticity at a synapse is bidirectional, resulting in either long-term potentiation (LTP) or long-term depression (LTD), and that the threshold for induction of LTP versus LTD of synapses is not stable but dynamic (Bienenstock et al., 1982; Murakami et al., 2012). The BCM model states that prior excitation will elevate the excitation threshold and thus decrease the predisposition for excitation, whereas prior inhibition will lower the excitation threshold and thus increase the predisposition for excitation (Murakami et al., 2012; Hurley and Machado, 2017). Therefore, as both tDCS and treadmill walking increase cortical excitability, performing DTW after tDCS + STW may facilitate LTD (decreased M1 activity).

We did not observe statistical differences in PFC ΔHbO_2_, gait parameters or in the cognitive task following active tDCS + STW intervention. There are several possible explanations. Firstly, we have used a small electrode size (3 cm × 3 cm). A small electrode allows stimulating a more focal area while a large anodal electrode targets a more widespread region (Bikson et al., 2013; Thair et al., 2017). Stimulating not only the area underlying the anodal electrode, but also surrounding areas within the regions may enhance the tDCS benefits. For example, Chen and Machado (2017) using a 3 cm × 3 cm anodal electrode did not show benefits on saccadic eye movement behavior, but Chen et al. (2018) showed improvements in oculomotor control following tDCS using 5 cm × 7 cm anodal electrode. Secondly, the stimulation intensity may have been too low. We applied 0.6 mA, whereas most studies have used either 1.0 or 2.0 mA (de Paz et al., 2019; Beretta et al., 2020). We selected this intensity to ensure the current density was within the recommended safety limit of 0.029–0.08 mA/cm^2^ (Nitsche et al., 2008). The surface area of our anodal electrodes was 9 cm^2^ resulting in a current density of 0.067 mA/cm^2^. Several studies that used 1–2 mA also reported inconclusive results (de Paz et al., 2019; Beretta et al., 2020). While some studies have shown positive effects of stimulation combined with training in gait parameters (Kaski et al., 2014b,a; Park et al., 2015; Manenti et al., 2016; Ishikuro et al., 2018), others have not (Costa-Ribeiro et al., 2016, 2017; Kumru et al., 2016; Manenti et al., 2016; Fernández-Lago et al., 2017; Seo et al., 2017; Yotnuengnit et al., 2018). Although OA decreased stride time variability in the post-compared to pre-intervention, this was not related to active tDCS + STW, since both sham and active interventions presented a reduction. This indicates a training effect for OA, who are generally less familiar with treadmill walking than YA. Thirdly, studies that observed positive effects of anodal tDCS combined with training have investigated patients with neurological disorders, such as Parkinson’s disease and stroke (de Paz et al., 2019; Beretta et al., 2020). Fourthly, the lack of change in gait parameters following active tDCS + STW may be explained by the physically active participants who all performed more than 150 min of physical activity per week. A single session of 20 min treadmill walking combined with low current tDCS may therefore not be sufficient to induce gait changes (Silva et al., 2019). Specifically, regarding the cognitive task, a possible reason for no observed improvement could be the high cognitive functionality of the OA group (no difference in MoCA between age groups) and the floor effect (participants presented a low percentage of errors in the cognitive digit vigilance task – Table 2), reducing the amount participants could improve. We recommend the cognitive task is standardized by age group in future studies (de Rond et al., 2021). Taken together, further investigation is necessary to optimize tDCS protocols in gait rehabilitation.

Although the tDCS + STW intervention was not found to increase PFC HbO_2_, within the active tDCS groups, partial correlation showed that higher increases in PFC activity were associated with greater decreases in stride length variability. Our data also showed that OA presented higher activity bilaterally in the PFC and M1, and unilaterally in the right PMC compared to YA, which may reflect a mechanism to compensate for age-related decrease in gait automaticity (Stuart et al., 2018; Al-Yahya et al., 2019). In addition, OA presented higher gait variability than YA, which suggests reduced movement automaticity (Hausdorff et al., 2001; Verghese et al., 2009; Sawa et al., 2014; Aboutorabi et al., 2016). Taken together, these findings may indicate that the combined intervention could expand the availability of prefrontal executive-attentional resources to be allocated to the control of walking, leading to better movement automaticity (da Conceição et al., 2021).

A key strength of this study is the sham protocol, which was effective in blinding participants to the tDCS condition. Indeed, we did not observe a difference in the adverse events questionnaire between the sham and active groups, which confirms the participant blinding. Also, the concurrent assessment of multiple cortical areas (PFC, PMC, SMA, and M1) while walking together with gait parameters provides better understanding of aspects involved in the gait control and the potential mechanisms underlying gait improvements obtained with the combined intervention. However, this study presents some limitations. The small sample size is an important limitation resulting in low statistical power and may account for the lack of significant change in fNIRS signals, gait parameters and cognitive tasks following anodal tDCS. The small sample size may also have prevented us from finding other associations between changes in cortical activity and gait parameters. The absence of a control group who did not perform any of the intervention protocols or an isolated tDCS session limits our interpretations. The study only involved a single session rather than a series of sessions which may have provided significant longitudinal results (El-Sayes et al., 2019). The number of women and men in the groups was unbalanced. Previous studies have reported sex-specific cortical activation, which suggests that sex may affect fNIRS signals (Leon-Carrion et al., 2006; Li et al., 2010; Baker et al., 2016). Another limitation concerns the treadmill task. Previous studies have reported a significant difference in hemodynamic data when individuals walked on a treadmill compared to overground (Clark et al., 2014; Thumm et al., 2018) due to a treadmill acting as an external regulator of gait (Suzuki et al., 2004; Harada et al., 2009). There are limitations in recording fNIRS signals as we did not use short-separation channels to control for scalp blood flow. However, we applied Wavelet-MDL detrending to remove unknown global trends from our data, which has been shown to be acceptable (Jang et al., 2009; Herold et al., 2017; Vitorio et al., 2017). Another limitation is the use of a subjective scale (Borg) to assess exercise intensity. Although the Borg scale is a valid tool for monitoring exercise intensity (Scherr et al., 2013), an objective physiological measure (e.g., heart rate) would be more precise in order to ensure that all four groups experienced the similar intensity of walking (Chen et al., 2002). A further limitation is that the older adults who frequently volunteer for studies are often physically more active and cognitively higher functioning than is typical for their age group, which may have lessened the chances of the older adults benefiting from the active combined intervention due to less room for improvement relative to the general population. Therefore, we recommend addressing these limitations in future studies.

In conclusion, an intervention using anodal tDCS applied to both PFC and M1 cortical regions combined with STW decreased M1 cortical activity during DTW in both YA and OA. As gait parameters remained unchanged, this suggests an improvement in neural efficiency. In addition, higher increases in PFC activity after combined tDCS + STW intervention is related to better gait automaticity.

## Data Availability Statement

The raw data supporting the conclusions of this article will be made available by the authors, without undue reservation.

## Ethics Statement

The studies involving human participants were reviewed and approved by Newcastle University Faculty of Medical Sciences Ethics Committee (Ref. 6770/2018). The patients/participants provided their written informed consent to participate in this study.

## Author Contributions

DO-S designed the study, collected, analyzed, and interpreted the data, and drafted the manuscript for intellectual content. AP designed and conceptualized the study, interpreted the data, and revised the manuscript for intellectual content. AI designed the study, collected, analyzed, and interpreted the data, and revised the manuscript for intellectual content. LTBG and LR interpreted the data and revised the manuscript for intellectual content. MB designed the study, interpreted the data, and revised the manuscript for intellectual content. All authors approved the final manuscript.

## Conflict of Interest

The authors declare that the research was conducted in the absence of any commercial or financial relationships that could be construed as a potential conflict of interest.

## Publisher’s Note

All claims expressed in this article are solely those of the authors and do not necessarily represent those of their affiliated organizations, or those of the publisher, the editors and the reviewers. Any product that may be evaluated in this article, or claim that may be made by its manufacturer, is not guaranteed or endorsed by the publisher.

## References

[B1] Aboutorabi A. Arazpour M. Bahramizadeh M. Hutchins S. W. Fadayevatan R. (2016). The effect of aging on gait parameters in able-bodied older subjects: a literature review. *Aging Clin. Exp. Res.* 28 393–405. 10.1007/s40520-015-0420-6 26210370

[B2] Al-Yahya E. Dawes H. Smith L. Dennis A. Howells K. Cockburn J. (2011). Cognitive motor interference while walking: a systematic review and meta-analysis. *Neurosci. Biobehav. Rev.* 35 715–728. 10.1016/j.neubiorev.2010.08.008 20833198

[B3] Al-Yahya E. Mahmoud W. Meester D. Esser P. Dawes H. (2019). Neural substrates of cognitive motor interference during walking; peripheral and central mechanisms. *Front. Hum. Neurosci.* 12:536. 10.3389/fnhum.2018.00536 30687049 PMC6333849

[B4] Andrews S. C. Hoy K. E. Enticott P. G. Daskalakis Z. J. Fitzgerald P. B. (2011). Improving working memory: the effect of combining cognitive activity and anodal transcranial direct current stimulation to the left dorsolateral prefrontal cortex. *Brain Stimul.* 4 84–89. 10.1016/j.brs.2010.06.004 21511208

[B5] Baker J. M. Liu N. Cui X. Vrticka P. Saggar M. Hosseini S. M. H. (2016). Sex differences in neural and behavioral signatures of cooperation revealed by fNIRS hyperscanning. *Sci. Rep.* 6:26492. 10.1038/srep26492 27270754 PMC4897646

[B6] Beck O. N. Gosyne J. Franz J. R. Sawicki G. S. (2020). Cyclically producing the same average muscle-tendon force with a smaller duty increases metabolic rate. *Proc. R. Soc. B Biol. Sci.* 287:20210012. 10.1098/rspb.2020.0431 32811308 PMC7482283

[B7] Beretta V. S. Conceição N. R. Nóbrega-Sousa P. Orcioli-Silva D. Dantas L. K. B. F. Gobbi L. T. B. (2020). Transcranial direct current stimulation combined with physical or cognitive training in people with Parkinson’s disease: a systematic review. *J. Neuroeng. Rehabil.* 17:74. 10.1186/s12984-020-00701-6 32539819 PMC7296764

[B8] Beurskens R. Bock O. (2012). Age-related deficits of dual-task walking: a review. *Neural Plast.* 2012:131608. 10.1155/2012/131608 22848845 PMC3403123

[B9] Bienenstock E. Cooper L. Munro P. (1982). Theory for the development of neuron selectivity: orientation specificity and binocular interaction in visual cortex. *J. Neurosci.* 2 32–48. 10.1523/JNEUROSCI.02-01-00032.1982 7054394 PMC6564292

[B10] Bierre K. L. Lucas S. J. E. Guiney H. Cotter J. D. Machado L. (2017). Cognitive difficulty intensifies age-related changes in anterior frontal hemodynamics: novel evidence from near-infrared spectroscopy. *J. Gerontol. Ser. A Biol. Sci. Med. Sci.* 72 181–188. 10.1093/gerona/glw061 27048517

[B11] Bikson M. Name A. Rahman A. (2013). Origins of specificity during tDCS: anatomical, activity-selective, and input-bias mechanisms. *Front. Hum. Neurosci.* 7:688. 10.3389/fnhum.2013.00688 24155708 PMC3800813

[B12] Brunoni A. R. Amadera J. Berbel B. Volz M. S. Rizzerio B. G. Fregni F. (2011). A systematic review on reporting and assessment of adverse effects associated with transcranial direct current stimulation. *Int. J. Neuropsychopharmacol.* 14 1133–1145. 10.1017/S1461145710001690 21320389

[B13] Brunoni A. R. Nitsche M. A. Bolognini N. Bikson M. Wagner T. Merabet L. (2012). Clinical research with transcranial direct current stimulation (tDCS): challenges and future directions. *Brain Stimul.* 5 175–195. 10.1016/j.brs.2011.03.002 22037126 PMC3270156

[B14] Byun K. Hyodo K. Suwabe K. Ochi G. Sakairi Y. Kato M. (2014). Positive effect of acute mild exercise on executive function *via* arousal-related prefrontal activations: an fNIRS study. *NeuroImage* 98 336–345. 10.1016/j.neuroimage.2014.04.067 24799137

[B15] Cabeza R. Anderson N. D. Locantore J. K. McIntosh A. R. (2002). Aging gracefully: compensatory brain activity in high-performing older adults. *NeuroImage* 17 1394–1402. 10.1006/nimg.2002.1280 12414279

[B16] Chen M. J. Fan X. Moe S. T. (2002). Criterion-related validity of the Borg ratings of perceived exertion scale in healthy individuals: a meta-analysis. *J. Sports Sci.* 20 873–899. 10.1080/026404102320761787 12430990

[B17] Chen P. Stenling A. Machado L. (2018). Evidence transcranial direct current stimulation can improve saccadic eye movement control in older adults. *Vision* 2:42. 10.3390/vision2040042 31735905 PMC6835567

[B18] Chen P. L. Machado L. (2017). Developing clinically practical transcranial direct current stimulation protocols to improve saccadic eye movement control. *J. Eye Mov. Res.* 10. 10.16910/jemr.10.3.5 33828658 PMC7141088

[B19] Chrysikou E. G. Hamilton R. H. Coslett H. B. Datta A. Bikson M. Thompson-Schill S. L. (2013). Noninvasive transcranial direct current stimulation over the left prefrontal cortex facilitates cognitive flexibility in tool use. *Cogn. Neurosci.* 4 81–89. 10.1080/17588928.2013.768221 23894253 PMC3719984

[B20] Clark D. J. Christou E. A. Ring S. A. Williamson J. B. Doty L. (2014). Enhanced somatosensory feedback reduces prefrontal cortical activity during walking in older adults. *J. Gerontol. Ser. A Biol. Sci. Med. Sci.* 69 1422–1428. 10.1093/gerona/glu125 25112494 PMC4229993

[B21] Costa-Ribeiro A. Maux A. Bosford T. Aoki Y. Castro R. Baltar A. (2017). Transcranial direct current stimulation associated with gait training in Parkinson’s disease: a pilot randomized clinical trial. *Dev. Neurorehabil.* 20 121–128. 10.3109/17518423.2015.1131755 26864140

[B22] Costa-Ribeiro A. Maux A. Bosford T. Tenório Y. Marques D. Carneiro M. (2016). Dopamine-independent effects of combining transcranial direct current stimulation with cued gait training on cortical excitability and functional mobility in Parkinson’s disease. *J. Rehabil. Med.* 48 819–823. 10.2340/16501977-2134 27608611

[B23] da Conceição N. R. Gobbi L. T. B. Nóbrega-Sousa P. Orcioli-Silva D. Beretta V. S. Lirani-Silva E. (2021). Aerobic Exercise combined with transcranial direct current stimulation over the prefrontal cortex in Parkinson disease: effects on cortical activity, gait, and cognition. *Neurorehabil. Neural Rep.* 35 717–728. 10.1177/15459683211019344 34047235

[B24] de Paz R. H. Serrano-Muñoz D. Pérez-Nombela S. Bravo-Esteban E. Avendaño-Coy J. Gómez-Soriano J. (2019). Combining transcranial direct-current stimulation with gait training in patients with neurological disorders: a systematic review. *J. NeuroEng. Rehabil.* 16:114. 10.1186/s12984-019-0591-z 31521179 PMC6744683

[B25] de Rond V. Orcioli-Silva D. Dijkstra B. W. Orban de Xivry J.-J. Pantall A. Nieuwboer A. (2021). Compromised brain activity with age during a game-like dynamic balance task: single- vs. dual-task performance. *Front. Aging Neurosci.* 13:657308. 10.3389/fnagi.2021.657308 34290599 PMC8287632

[B26] Del Din S. Godfrey A. Rochester L. (2016). Validation of an accelerometer to quantify a comprehensive battery of gait characteristics in healthy older adults and Parkinson’s disease: toward clinical and at home use. *IEEE J. Biomed. Health Inform.* 20 838–847. 10.1109/JBHI.2015.2419317 25850097

[B27] El-Sayes J. Harasym D. Turco C. V. Locke M. B. Nelson A. J. (2019). Exercise-induced neuroplasticity: a mechanistic model and prospects for promoting plasticity. *Neuroscientist* 25 65–85. 10.1177/1073858418771538 29683026

[B28] Fernández-Lago H. Bello O. Mora-Cerdá F. Montero-Cámara J. Fernández-Del-Olmo M. Á (2017). Treadmill walking combined with anodal transcranial direct current stimulation in parkinson disease: a pilot study of kinematic and neurophysiological effects. *Am. J. Phys. Med. Rehabil.* 96 801–808. 10.1097/PHM.0000000000000751 28398968

[B29] Fregni F. Boggio P. S. Nitsche M. Bermpohl F. Antal A. Feredoes E. (2005). Anodal transcranial direct current stimulation of prefrontal cortex enhances working memory. *Exp. Brain Res.* 166 23–30. 10.1007/s00221-005-2334-6 15999258

[B30] Friston K. J. Josephs O. Zarahn E. Holmes A. P. Rouquette S. Poline J. B. (2000). To smooth or not to smooth? Bias and efficiency in fMRI time-series analysis. *NeuroImage* 12 196–208. 10.1006/nimg.2000.0609 10913325

[B31] Harada T. Miyai I. Suzuki M. Kubota K. (2009). Gait capacity affects cortical activation patterns related to speed control in the elderly. *Exp. Brain Res.* 193 445–454. 10.1007/s00221-008-1643-y 19030850

[B32] Hausdorff J. M. Rios D. A. Edelberg H. K. (2001). Gait variability and fall risk in community-living older adults: a 1-year prospective study. *Arch. Phys. Med. Rehabil.* 82 1050–1056. 10.1053/apmr.2001.24893 11494184

[B33] Hausdorff J. M. Schweiger A. Herman T. Yogev-Seligmann G. Giladi N. (2008). Dual-task decrements in gait: contributing factors among healthy older adults. *J. Gerontol. Ser. A Biol. Sci. Med. Sci.* 63 1335–1343. 10.1093/gerona/63.12.1335 19126846 PMC3181497

[B34] Heinrich S. Rapp K. Rissmann U. Becker C. König H.-H. (2009). Cost of falls in old age: a systematic review. *Osteoporos. Intl*. 21 891–902. 10.1007/s00198-009-1100-1 19924496

[B35] Herold F. Wiegel P. Scholkmann F. Thiers A. Hamacher D. Schega L. (2017). Functional near-infrared spectroscopy in movement science: a systematic review on cortical activity in postural and walking tasks. *Neurophotonics* 4:41403. 10.1117/1.NPh.4.4.041403 PMC5538329 28924563

[B36] Hurley R. Machado L. (2017). Using tDCS priming to improve brain function: can metaplasticity provide the key to boosting outcomes? *Neurosci. Biobehav. Rev.* 83 155–159. 10.1016/j.neubiorev.2017.09.029 29020606

[B37] Hurley R. Machado L. (2018). Using transcranial direct current stimulation to improve verbal working memory: a detailed review of the methodology. *J. Clin. Exp. Neuropsychol.* 40 790–804. 10.1080/13803395.2018.1434133 29429396

[B38] Ishikuro K. Dougu N. Nukui T. Yamamoto M. Nakatsuji Y. Kuroda S. (2018). Effects of transcranial direct current stimulation (tDCS) over the frontal polar area on motor and executive functions in Parkinson’s disease; a pilot study. *Front. Aging Neurosci.* 10:231. 10.3389/fnagi.2018.00231 30104971 PMC6077209

[B39] Jang K. E. Tak S. Jung J. Jang J. Jeong Y. Ye J. C. (2009). Wavelet minimum description length detrending for near-infrared spectroscopy. *J. Biomed. Opt.* 14:034004. 10.1117/1.3127204 19566297

[B40] Javadi A. H. Walsh V. (2012). Transcranial direct current stimulation (tDCS) of the left dorsolateral prefrontal cortex modulates declarative memory. *Brain Stimul.* 5 231–241. 10.1016/j.brs.2011.06.007 21840287

[B41] Ji Z. Feng T. Mei L. Li A. Zhang C. (2019). Influence of acute combined physical and cognitive exercise on cognitive function: an NIRS study. *PeerJ* 7:e7418. 10.7717/peerj.7418 31396453 PMC6681798

[B42] Kaski D. Allum J. H. Bronstein A. M. Dominguez R. O. (2014a). Applying anodal tDCS during tango dancing in a patient with Parkinson’s disease. *Neurosci. Lett.* 568 39–43. 10.1016/j.neulet.2014.03.043 24686184

[B43] Kaski D. Dominguez R. O. Allum J. H. Islam A. F. Bronstein A. M. (2014b). Combining physical training with transcranial direct current stimulation to improve gait in Parkinson’s disease: a pilot randomized controlled study. *Clin. Rehabil.* 28 1115–1124. 10.1177/0269215514534277 24849794

[B44] Koenraadt K. L. M. Roelofsen E. G. J. Duysens J. Keijsers N. L. W. (2014). Cortical control of normal gait and precision stepping: an fNIRS study. *NeuroImage* 85(Pt 1) 415–422. 10.1016/j.neuroimage.2013.04.070 23631980

[B45] Koganemaru S. Mikami Y. Maezawa H. Matsuhashi M. Ikeda S. Ikoma K. (2018). Anodal transcranial patterned stimulation of the motor cortex during gait can induce activity-dependent corticospinal plasticity to alter human gait. *PLoS One* 13:e0208691. 10.1371/journal.pone.0208691 30576315 PMC6303011

[B46] Kumru H. Murillo N. Benito-Penalva J. Tormos J. M. Vidal J. (2016). Transcranial direct current stimulation is not effective in the motor strength and gait recovery following motor incomplete spinal cord injury during Lokomat(§) gait training. *Neurosci. Lett.* 620 143–147. 10.1016/j.neulet.2016.03.056 27040426

[B47] la Fougère C. Zwergal A. Rominger A. Förster S. Fesl G. Dieterich M. (2010). Real versus imagined locomotion: a [18F]-FDG PET-fMRI comparison. *NeuroImage* 50 1589–1598. 10.1016/j.neuroimage.2009.12.060 20034578

[B48] Leon-Carrion J. Damas J. Izzetoglu K. Pourrezai K. Martín-Rodríguez J. F. Barroso y Martin J. M. (2006). Differential time course and intensity of PFC activation for men and women in response to emotional stimuli: a functional near-infrared spectroscopy (fNIRS) study. *Neurosci. Lett.* 403 90–95. 10.1016/j.neulet.2006.04.050 16716510

[B49] Li T. Luo Q. Gong H. (2010). Gender-specific hemodynamics in prefrontal cortex during a verbal working memory task by near-infrared spectroscopy. *Behav. Brain Res.* 209 148–153. 10.1016/j.bbr.2010.01.033 20117145

[B50] Lin S.-I. Chang K.-C. Lee H.-C. Yang Y.-C. Tsauo J.-Y. (2015). Problems and fall risk determinants of quality of life in older adults with increased risk of falling. *Geriatr. Gerontol. Int.* 15 579–587. 10.1111/ggi.12320 25109554

[B51] Lord S. R. Lloyd D. G. Keung Li S. E. K. (1996). Sensori-motor function, gait patterns and falls in community-dwelling women. *Age Ageing* 25 292–299. 10.1093/ageing/25.4.292 8831874

[B52] Machado L. (2021). Understanding cognition and how it changes with aging, brain disease, and lifestyle choices. *J. R. Soc. N. Z.* 51 128–142. 10.1080/03036758.2020.1796102

[B53] Maidan I. Nieuwhof F. Bernad-Elazari H. Reelick M. F. Bloem B. R. Giladi N. (2016). the role of the frontal lobe in complex walking among patients with Parkinson’s disease and healthy older adults: an fNIRS study. *Neurorehabil. Neural Rep.* 30 963–971. 10.1177/1545968316650426 27221042

[B54] Manenti R. Brambilla M. Benussi A. Rosini S. Cobelli C. Ferrari C. (2016). Mild cognitive impairment in Parkinson’s disease is improved by transcranial direct current stimulation combined with physical therapy. *Mov. Disord.* 31 715–724. 10.1002/mds.26561 26880536

[B55] Manor B. Zhou J. Harrison R. Lo O.-Y. Travison T. G. Hausdorff J. M. (2018). Transcranial direct current stimulation may improve cognitive-motor function in functionally limited older adults. *Neurorehabil. Neural Rep.* 32 788–798. 10.1177/1545968318792616 30132389 PMC6143414

[B56] McCamley J. Donati M. Grimpampi E. Mazza C. (2012). An enhanced estimate of initial contact and final contact instants of time using lower trunk inertial sensor data. *Gait Posture* 36 316–318. 10.1016/j.gaitpost.2012.02.019 22465705

[B57] Mirelman A. Maidan I. Bernad-Elazari H. Shustack S. Giladi N. Hausdorff J. M. (2017). Effects of aging on prefrontal brain activation during challenging walking conditions. *Brain Cogn.* 115 41–46. 10.1016/j.bandc.2017.04.002 28433922

[B58] Moe-Nilssen R. (1998). A new method for evaluating motor control in gait under real-life environmental conditions. Part 1: the instrument. *Clin. Biomech. (Bristol, Avon)* 13 320–327. 10.1016/s0268-0033(98)00089-8 11415803

[B59] Morris R. Lord S. Bunce J. Burn D. Rochester L. (2016). Gait and cognition: mapping the global and discrete relationships in ageing and neurodegenerative disease. *Neurosci. Biobehav. Rev.* 64 326–345. 10.1016/j.neubiorev.2016.02.012 26915926

[B60] Murakami T. Müller-Dahlhaus F. Lu M.-K. Ziemann U. (2012). Homeostatic metaplasticity of corticospinal excitatory and intracortical inhibitory neural circuits in human motor cortex. *J. Physiol.* 590 5765–5781. 10.1113/jphysiol.2012.238519 22930265 PMC3528990

[B61] Murray L. M. Edwards D. J. Ruffini G. Labar D. Stampas A. Pascual-Leone A. (2015). Intensity dependent effects of transcranial direct current stimulation on corticospinal excitability in chronic spinal cord injury. *Arch. Phys. Med. Rehabil.* 96 S114–S121. 10.1016/j.apmr.2014.11.004 25461825 PMC4380548

[B62] Nasreddine Z. S. Phillips N. A. Bedirian V. Charbonneau S. Whitehead V. Collin I. (2005). The montreal cognitive assessment, MoCA: a brief screening tool for mild cognitive impairment. *J. Am. Geriatr. Soc.* 53 695–699. 10.1111/j.1532-5415.2005.53221.x 15817019

[B63] Nitsche M. A. Cohen L. G. Wassermann E. M. Priori A. Lang N. Antal A. (2008). Transcranial direct current stimulation: state of the art 2008. *Brain Stimul.* 1 206–223. 10.1016/j.brs.2008.06.004 20633386

[B64] Nitsche M. A. Paulus W. (2000). Excitability changes induced in the human motor cortex by weak transcranial direct current stimulation. *J. Physiol.* 527(Pt 3) 633–639. 10.1111/j.1469-7793.2000.t01-1-00633.x 10990547 PMC2270099

[B65] Nóbrega-Sousa P. Gobbi L. T. B. Orcioli-Silva D. Conceição N. R. Beretta V. S. Vitório R. (2020). Prefrontal cortex activity during walking: effects of aging and associations with gait and executive function. *Neurorehabil. Neural Rep.* 34 915–924. 10.1177/1545968320953824 32865134

[B66] Orcioli-Silva D. Vitório R. Beretta V. S. da Conceição N. R. Nóbrega-Sousa P. Oliveira A. S. (2021). Is cortical activation during walking different between Parkinson’s disease motor subtypes? *J. Gerontol. Ser. A* 76 561–567. 10.1093/gerona/glaa174 32674140

[B67] Orcioli-Silva D. Vitório R. Nóbrega-Sousa P. da Conceição N. R. Beretta V. S. Lirani-Silva E. (2020). Levodopa facilitates prefrontal cortex activation during dual task walking in Parkinson disease. *Neurorehabil. Neural Rep.* 34 589–599. 10.1177/1545968320924430 32449460

[B68] Park S. D. Kim J. Y. Song H. S. (2015). Effect of application of transcranial direct current stimulation during task-related training on gait ability of patients with stroke. *J. Phys. Ther. Sci.* 27 623–625. 10.1589/jpts.27.623 25931694 PMC4395678

[B69] Pelicioni P. H. S. Tijsma M. Lord S. R. Menant J. (2019). Prefrontal cortical activation measured by fNIRS during walking: effects of age, disease and secondary task. *PeerJ* 7:e6833. 10.7717/peerj.6833 31110922 PMC6501770

[B70] Petersen N. T. Butler J. E. Marchand-Pauvert V. Fisher R. Ledebt A. Pyndt H. S. (2001). Suppression of EMG activity by transcranial magnetic stimulation in human subjects during walking. *J. Physiol.* 537 651–656. 10.1111/j.1469-7793.2001.00651.x 11731595 PMC2278954

[B71] Petersen T. H. Willerslev-Olsen M. Conway B. A. Nielsen J. B. (2012). The motor cortex drives the muscles during walking in human subjects. *J. Physiol.* 590 2443–2452. 10.1113/jphysiol.2012.227397 22393252 PMC3424763

[B72] Plummer-D’Amato P. Brancato B. Dantowitz M. Birken S. Bonke C. Furey E. (2012). Effects of gait and cognitive task difficulty on cognitive-motor interference in aging. *J. Aging Res.* 2012:583894. 10.1155/2012/583894 23209905 PMC3503314

[B73] Sawa R. Doi T. Misu S. Tsutsumimoto K. Nakakubo S. Asai T. (2014). The association between fear of falling and gait variability in both leg and trunk movements. *Gait Posture* 40 123–127. 10.1016/j.gaitpost.2014.03.002 24656714

[B74] Scherr J. Wolfarth B. Christle J. W. Pressler A. Wagenpfeil S. Halle M. (2013). Associations between Borg’s rating of perceived exertion and physiological measures of exercise intensity. *Eur. J. Appl. Physiol.* 113 147–155. 10.1007/s00421-012-2421-x 22615009

[B75] Schneider N. Dagan M. Katz R. Thumm P. C. Brozgol M. Giladi N. (2021). Combining transcranial direct current stimulation with a motor-cognitive task: the impact on dual-task walking costs in older adults. *J. NeuroEng. Rehabil.* 18:23. 10.1186/s12984-021-00826-2 33526043 PMC7852224

[B76] Seo H. G. Lee W. H. Lee S. H. Yi Y. Kim K. D. Oh B.-M. (2017). Robotic-assisted gait training combined with transcranial direct current stimulation in chronic stroke patients: a pilot double-blind, randomized controlled trial. *Restor. Neurol. Neurosci.* 35 527–536. 10.3233/RNN-170745 28800341

[B77] Silva L. V. D. C. Porto F. Fregni F. Gurgel J. L. (2019). Transcranial direct-current stimulation in combination with exercise: a systematic review. *Rev. Brasil. Med. Esporte* 25 520–526.

[B78] Steinberg F. Pixa N. H. Fregni F. (2018). A review of acute aerobic exercise and transcranial direct current stimulation effects on cognitive functions and their potential synergies. *Front. Hum. Neurosci.* 12:534. 10.3389/fnhum.2018.00534 30687048 PMC6336823

[B79] Stuart S. Vitorio R. Morris R. Martini D. N. Fino P. C. Mancini M. (2018). Cortical activity during walking and balance tasks in older adults and in people with Parkinson’s disease: a structured review. *Maturitas* 113 53–72. 10.1016/j.maturitas.2018.04.011 29903649 PMC6448561

[B80] Studenski S. Perera S. Patel K. Rosano C. Faulkner K. Inzitari M. (2011). Gait speed and survival in older adults. *JAMA* 305 50–58. 10.1001/jama.2010.1923 21205966 PMC3080184

[B81] Suzuki M. Miyai I. Ono T. Oda I. Konishi I. Kochiyama T. (2004). Prefrontal and premotor cortices are involved in adapting walking and running speed on the treadmill: an optical imaging study. *NeuroImage* 23 1020–1026. 10.1016/j.neuroimage.2004.07.002 15528102

[B82] Thair H. Holloway A. L. Newport R. Smith A. D. (2017). Transcranial direct current stimulation (tDCS): a beginner’s guide for design and implementation. *Front. Neurosci.* 11:641. 10.3389/fnins.2017.00641 29213226 PMC5702643

[B83] Thumm P. C. Maidan I. Brozgol M. Shustak S. Gazit E. Shema Shiratzki S. (2018). Treadmill walking reduces pre-frontal activation in patients with Parkinson’s disease. *Gait Post.* 62 384–387. 10.1016/j.gaitpost.2018.03.041 29626840

[B84] Tsuzuki D. Dan I. (2014). Spatial registration for functional near-infrared spectroscopy: from channel position on the scalp to cortical location in individual and group analyses. *NeuroImage* 85(Pt 1) 92–103. 10.1016/j.neuroimage.2013.07.025 23891905

[B85] Vanderhasselt M.-A. de Raedt R. Brunoni A. R. Campanhã C. Baeken C. Remue J. (2013). tDCS over the left prefrontal cortex enhances cognitive control for positive affective stimuli. *PLoS One* 8:e62219. 10.1371/journal.pone.0062219 23704874 PMC3660532

[B86] Verghese J. Holtzer R. Lipton R. B. Wang C. (2009). Quantitative gait markers and incident fall risk in older adults. *J. Gerontol. Ser. A Biol. Sci. Med. Sci.* 64 896–901. 10.1093/gerona/glp033 19349593 PMC2709543

[B87] Vitorio R. Stuart S. Gobbi L. T. B. Rochester L. Alcock L. Pantall A. (2018). Reduced gait variability and enhanced brain activity in older adults with auditory cues: a functional near-infrared spectroscopy study. *Neurorehabil. Neural Rep.* 32 976–987. 10.1177/1545968318805159 30411674

[B88] Vitorio R. Stuart S. Rochester L. Alcock L. Pantall A. (2017). fNIRS response during walking — Artefact or cortical activity? A systematic review. *Neurosci. Biobehav. Rev.* 83 160–172. 10.1016/j.neubiorev.2017.10.002 29017917

[B89] Voigt M. Hyttel M. K. Jakobsen L. S. Jensen M. K. Balle H. Hansen E. A. (2019). Human walk-to-run transition in the context of the behaviour of complex systems. *Hum. Mov. Sci.* 67:102509. 10.1016/j.humov.2019.102509 31415962

[B90] Wrightson J. G. Twomey R. Ross E. Z. Smeeton N. J. (2015). The effect of transcranial direct current stimulation on task processing and prioritisation during dual-task gait. *Exp. Brain Res.* 233 1575–1583. 10.1007/s00221-015-4232-x 25724513

[B91] Yanagisawa H. Dan I. Tsuzuki D. Kato M. Okamoto M. Kyutoku Y. (2010). Acute moderate exercise elicits increased dorsolateral prefrontal activation and improves cognitive performance with Stroop test. *NeuroImage* 50 1702–1710. 10.1016/j.neuroimage.2009.12.023 20006719

[B92] Yardley L. Beyer N. Hauer K. Kempen G. Piot-Ziegler C. Todd C. (2005). Development and initial validation of the falls efficacy scale-international (FES-I). *Age Ageing* 34 614–619. 10.1093/ageing/afi196 16267188

[B93] Ye J. C. Tak S. Jang K. E. Jung J. Jang J. (2009). NIRS-SPM: statistical parametric mapping for near-infrared spectroscopy. *NeuroImage* 44 428–447. 10.1016/j.neuroimage.2008.08.036 18848897

[B94] Yogev-Seligmann G. Hausdorff J. M. Giladi N. (2008). The role of executive function and attention in gait. *Mov. Disord.* 23 329–342. 10.1002/mds.21720 18058946 PMC2535903

[B95] Yotnuengnit P. Bhidayasiri R. Donkhan R. Chaluaysrimuang J. Piravej K. (2018). Effects of transcranial direct current stimulation plus physical therapy on gait in patients with Parkinson disease: a randomized controlled trial. *Am. J. Phys. Med. Rehabil.* 97 7–15. 10.1097/PHM.0000000000000783 28650857

[B96] Zarahn E. Rakitin B. Abela D. Flynn J. Stern Y. (2007). Age-related changes in brain activation during a delayed item recognition task. *Neurobiol. Aging* 28 784–798. 10.1016/j.neurobiolaging.2006.03.002 16621168

[B97] Zhou J. Hao Y. Wang Y. Jor’dan A. Pascual-Leone A. Zhang J. (2014). Transcranial direct current stimulation reduces the cost of performing a cognitive task on gait and postural control. *Eur. J. Neurosci.* 39 1343–1348. 10.1111/ejn.12492 24443958 PMC4221849

[B98] Zijlstra W. Hof A. L. (2003). Assessment of spatio-temporal gait parameters from trunk accelerations during human walking. *Gait Post.* 18 1–10. 10.1016/s0966-6362(02)00190-x 14654202

